# Spores of puffball fungus *Lycoperdon pyriforme* as a reference standard of stable monodisperse aerosol for calibration of optical instruments

**DOI:** 10.1371/journal.pone.0210754

**Published:** 2019-01-30

**Authors:** Anatoliy A. Zhirnov, Nina N. Kudryashova, Olga B. Kudryashova, Nataliya V. Korovina, Anatoliy A. Pavlenko, Sergey S. Titov

**Affiliations:** 1 Institute for Problems of Chemical and Energetic Technologies SB RAS, Biysk, Russia; 2 Life Science Center, Moscow Institute of Physics and Technology, Dolgoprudny city, Russia; 3 National Research Tomsk State University, Tomsk, Russia; VIT University, INDIA

## Abstract

Advanced air quality control requires real-time monitoring of particulate matter size and concentration, which can only be done using optical instruments. However, such techniques need regular calibration with reference samples. In this study, we suggest that puffball fungus (*Lycoperdon pyriforme*) spores can be utilized as a reference standard having a monodisperse size distribution. We compare the *Lycoperdon pyriforme* spores with the other commonly used reference samples, such as Al_2_O_3_ powder and polystyrene latex (PSL) microspheres. Here we demonstrate that the puffball spores do not coagulate and, thus, maintain the same particle size in the aerosol state for at least 15 minutes, which is enough for instrument calibration. Moreover, the puffball mushrooms can be stored for several years and no agglomeration of the spores occurs. They are also much cheaper than other calibration samples and no additional devices are needed for aerosol generation since the fungal fruiting body acts as an atomizer itself. The aforementioned features make the fungal spores a highly promising substance for calibration and validation of particle size analyzers, which outperforms the existing, artificially produced particles for aerosol sampling. Furthermore, the *L. pyriforme* spores are convenient for basic research and development of new optical measurement techniques, taking into account their uniform particle size and absent coagulation in the aerosol.

## Introduction

Air pollution is one of the major risk factors for public health. One of the components of air pollution both outdoors and indoors is fine particulate matter (PM). Micrometer-sized particles are especially hazardous, as they can infiltrate into biological tissues of lungs and blood vessels leading to cardiovascular disease [1, 2], respiratory disease [2, 3] and even cancer [4]. Microparticles are typically categorized into two groups: PM10 (10 *μ*m particles and smaller) and PM2.5 (2.5 *μ*m and smaller). The International Agency for Research on Cancer (IARC) of the World Health Organization (WHO) classifies these fine PM as a Group 1 carcinogen [5] and the WHO, therefore, encourages the reduction of fine particulates in the atmosphere.

The first essential step in controlling the air quality is to develop PM measurement methods [6]. A well established approach being used nowadays is based on weighing the filters containing trapped particles (gravimetric analysis), and has been accepted as a standard technique by the European Committee for Standardization (CEN) [7] and the National Institute of Standards and Technology (NIST) [8]. In addition, this approach is employed for biocontamination control in pharmaceutical production [9]. The gravimetric analysis is a direct and reliable method, which is, however, not suitable for real-time monitoring of the variations of PM concentrations and can only be applied to solid particulates rather than to liquid droplets [10]. Optical measurement techniques [11–13] are of use as an alternative, because they are free of the aforementioned limitations and can provide real-time solutions for measurement of particle concentrations and size distributions. There are many optical particle counters nowadays that solve most of the practical problems, such as detection of nanoparticulates at workplaces [14]. However, the particle counters measure only a total particulate concentration and do not distinguish particulate sizes that, in fact, affect the health in different ways [15, 16], and must therefore be identified for a proper air quality control. The optical techniques for measuring the concentration and size distribution of particulate matter require non-trivial calibration [10] and that is why they have not yet been accepted as standard methods [7].

Thus, the development of new optical instruments and the improvement of the existing ones for measuring solid and liquid particulate matter in aerosol state rely upon their calibration [17]. For this, reference standards with a certain particle size distribution are typically employed. Such standard samples may have a broad size distribution (polydisperse), for example, Arizona test dust (ISO 12103-1:2016) used in the US, or aluminum oxide powder [18] recommended by a standardization body in Russia. Otherwise, such samples may contain only one type of particles with a fixed size, i.e. highly monodisperse aerosols. Polystyrene latex (PSL) microspheres are most widely used to produce such monodisperse calibration samples [19]. Monodisperse samples are also often required in basic research on aerosols [20]. In practical applications, they also help avoid many difficulties and misinterpretations of the results, which could have been caused, for instance, by refraction of light on differently-sized particles. Thus, monodisperse aerosols facilitate the calibration process and allow for a better calibration accuracy of optical instruments [19, 21].

However, the generation of a highly monodisperse aerosol is a challenging problem. Many research teams have attempted to develop atomizers for this purpose [20, 22–24], since monodisperse aerosols are required in many basic [16] and applied research projects [19]. Such atomizers are only able to produce monodisperse liquid aerosols that lose the stability of the particle size distribution shortly after spraying due to rapid coagulation [25], droplet fragmentation and evaporation. Another approach being most widely used at present, is to atomize a water suspension of monodisperse PSL microspheres [19, 20, 26]. Yet, there are a few limitations to using PSL as a test aerosol sample because of its physical and chemical properties. Firstly, the same limitations as for liquid aerosols apply to this technique: water droplets carrying PSL particles are prone to coagulation and evaporation, which results in changing of the particle sizes over time [20]. Secondly, the atomization process produces numerous water droplets which do not contain PSL particles at all [27], but contribute to the particle size distribution. Thirdly, the PSL particles have a tendency to coagulate rapidly [28], impeding the aerosol measurement and instrument calibration. And finally, the size of PSL spheres changes during storage and can either decrease due to material irradiation or grow due to absorption of contaminants [19]. Consequently, the size can differ from that claimed by the manufacturer and this must be checked by an alternative method (such as SEM) prior to atomization. Hence, PSL particles cannot be used for research that requires a stable, highly monodisperse aerosol with a reliable particle size distribution, and there is currently no other experimental model that fully meets these requirements.

In this paper, we propose to use the puffball fungus spores of *Lycoperdon pyriforme* as a reference standard of monodisperse particles to calibrate optical instruments that measure aerosol particle sizes. We examine different monodisperse aerosols and demonstrate that the fungal spores are superior to the existing, artificially produced samples in the following aspects: no agglomeration occurs during long-term storage; no coagulation occurs in the aerosol state; the shape is spherical; particle size distribution in narrow (nearly monodisperse); the light absorption coefficient is high; and, finally, they are widely available and cheap. The rational key for these superb properties of the fungal aerosol lies in the fungal reproduction strategy, which favors and selects organisms that produce aerosols with a prolonged sedimentation time [29]. Thus, this study demonstrates how the *L. pyriforme* spores can be used in laboratory practice for basic research on aerosols as well as in practical measurements of aerosol characteristics.

## Materials and methods

### Particle size and morphology

The size distribution and shape of the puffball spores were measured in an OLYMPUS OMEC DC130 microscope. The fungal spores were dusted directly from the fungal fruiting body onto the microscope slide and secured with the cover slip. The Al_2_O_3_ were deposited on the slide and secured in a similar way, whereas latex microspheres were placed on the cover slip in water suspension. The images of the particles were acquired with a resolution of up to 0.7 *μm*. The data were analyzed using OLYMPUS Particle Image Processor (PIP 9.0) software. The distribution of primary particle sizes was characterized with the following set of parameters:

- mass mean particle diameter *D*(4, 3);
- arithmetic-mean particle diameter *D*(1, 0);
- median diameter *D*_50_;
- smallest particle diameter *D*_10_;
- largest particle diameter *D*_90_;
- specific surface area (S.S.A) [*m*^2^/*cm*^3^]

The measurement range was between 0.5 ÷ 3000 *μm*.

### Scanning electronic microscopy (SEM)

The puffball spores in the dry state were dusted directly from the fungal fruiting body onto the adhesive carbon tab (AGAR Scientific, UK). Latex microspheres stayed in the water suspension and were deposited by an ultrasonicator. The particles were then sputtered with 50 Å silver and examined in a scanning electronic microscope (SEM; JSM-840, JEON, Japan) with a 10 kV accelerating voltage.

### Particle size change over time

The time profile of the aerosol particle size distribution was studied with a Spraytec particle size analyzer (Malvern Instruments, UK https://www.malvernpanalytical.com/en/products/product-range/spraytec). The Spraytec measuring system relies on laser diffraction. The recorded scattering pattern was analyzed by an appropriate optical model, which was chosen depending on the particle size distribution and concentration. Such models are based on the Mie theory [30] and Fraunhofer approximation [31] with a patented multiple scattering correction [32]. The Spraytec has a maximum data acquisition rate of 10 kHz, with a size range from 0.1 ÷ 2000 *μm*.

### Aerosol generation

In experiments with the Al_2_O_3_ reference sample, a Craton SBG-01 air spray gun (China) was used to produce aerosols. In control experiments, the spores were extracted from the puffball fungus and weighted to document the total powder weight to be atomized in a test chamber.

**Fig 1:**
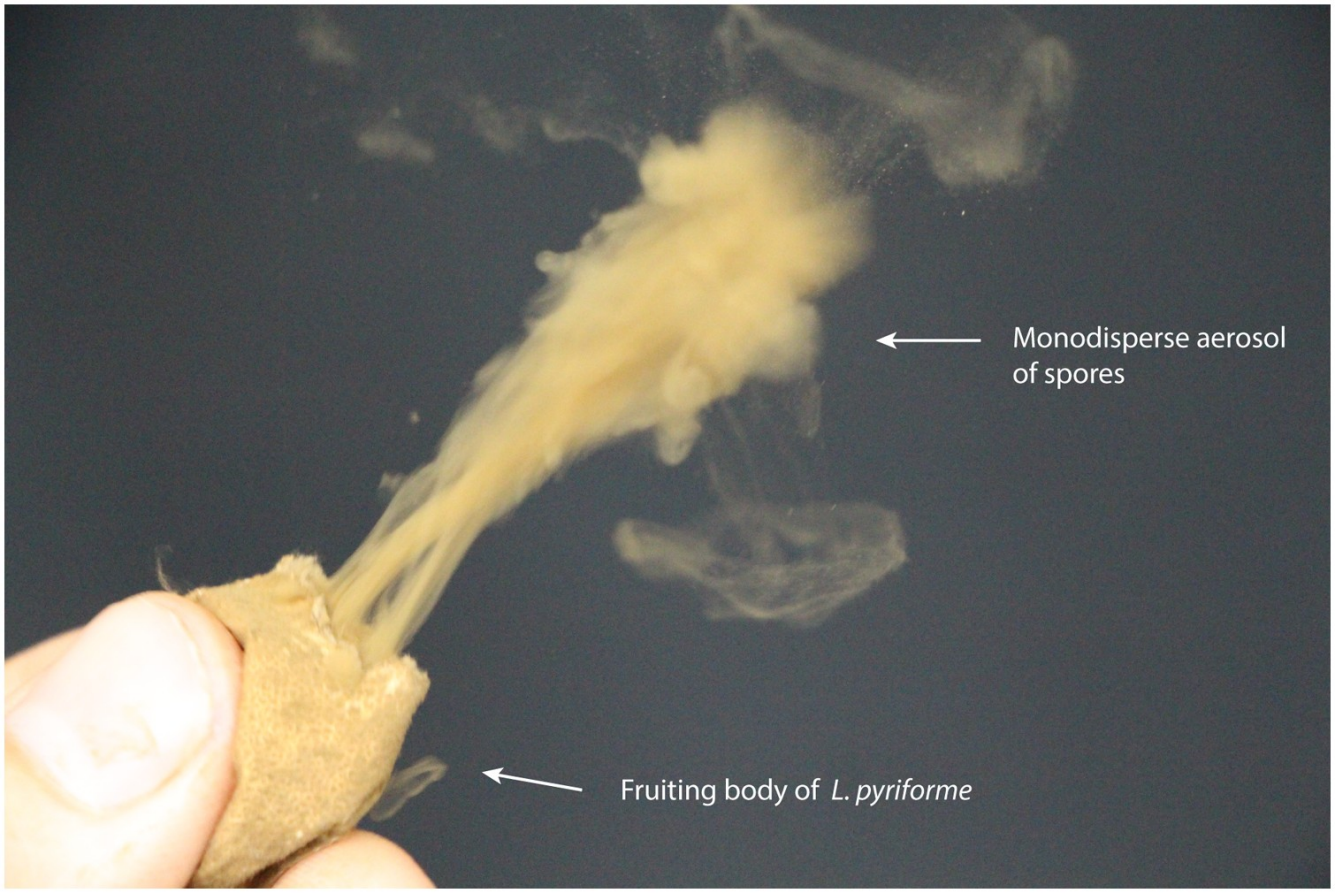
Puffball fungus *Lycoperdon pyriforme* sprays its spores. The fruiting body serves to generate an aerosol. In this figure, the puffball is dried-up as it has been stored for 2 years before the experiment, but this does not affect the aerosol characteristics, as shown by the data given hereafter.

The *L. pyriforme* spores were sprayed as described above or with the fungal fruiting body. Puffball fungi are natural “sprayers” (Fig 1), that produce an aerosol by spraying the spores through a small hole on the top of the fruiting body when squeezed. A footage of this spraying process is included in Supplementary Materials S1 Video.

The PSL suspension was atomized by a Mousson-2-03 sprayer. This sprayer generates a cloud of particles, 80% of which have a diameter below 4 *μm* (*D*_80_ = 4 *μm*).

## Results

In this section, the *L. pyriforme* spores are compared with the other two common aerosol reference standards chosen for a comparative purpose for the following reasons:

- polystyrene latex (PSL) microspheres are highly monodisperse and similar in morphology to the puffball spores;
- aluminum oxide powder is among widely used aerosol reference samples for calibration of optical instruments.

The systematic study into the properties of these three reference samples reveals principal distinctions between them and shows why the fungal spores are the best aerosol reference standard for many practical purposes.

### Size and morphology of standard samples

We have first examined the *L. pyriforme* spore size and morphology to compare them with the other reference standards of monodisperse aerosols. We used optical microscopy to measure the particle size distribution, and scanning electron microscopy (SEM) to observe particle morphology and their surface structure.

**Fig 2:**
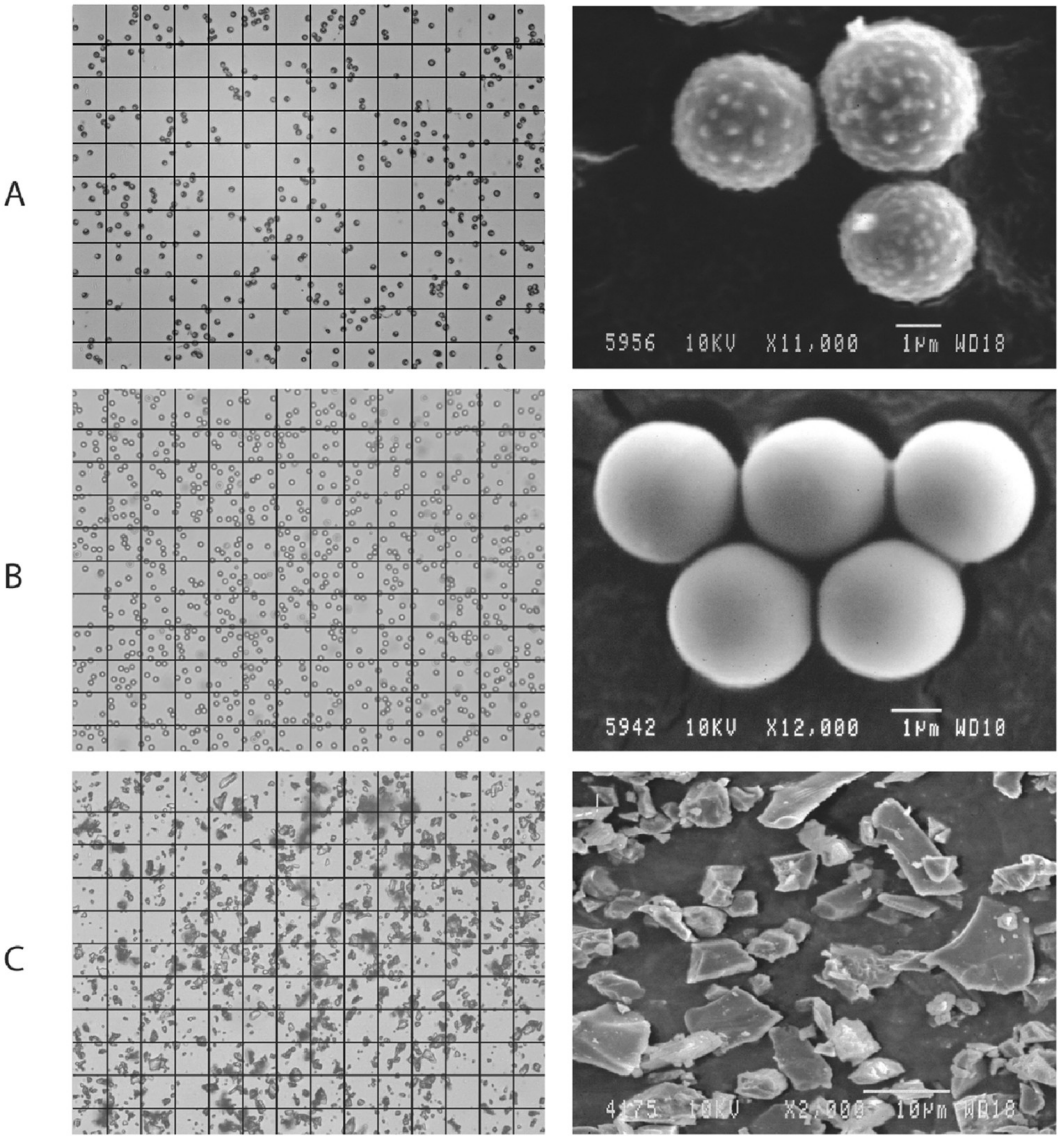
Morphological comparison of the particles of the three test samples. The left-hand images were taken at 400x zoom (grid size is 30 *μm*) with the optical microscope, and the right-hand images were taken with the SEM microscope and show the particles’ morphologies. (A) Spores of *L. pyriforme*. The image shows that the spores have a spherical shape and are almost identical in size. The SEM image shows a complex surface morphology of the spores. (B) Polystyrene latex (PSL) microspheres. Both images show that the all PSL spheres have a spherical shape and the same size. They are very stable in a water suspension, i.e. they do not form agglomerates therein (left-hand image) but coagulate quickly, once the solution dries up (see the “Long-term storage” section hereafter). (C) Al_2_O_3_ particles. The images show particles’ complex morphology and diverse sizes. Overall, their sizes are comparable to the PSL spheres and spores studied herein.

Fig 2A shows the *L. pyriforme* spores. The optical microscopy image (left) illustrates the uniformity of particle sizes with a resolution of 0.7 *μm*. The image was analyzed with OLYMPUS Particle Image Processor (PIP 9.0) software (measurements are available in S1 Table and example raw data is shown in S1 Appendix). The mean particle size varied from 3.0 to 3.4 *μm* between different mushrooms (being 3.3 *μm* on average), and the half-width of the particle size distribution was (*D*_90_ − *D*_10_)/2 ≈ 0.4 − 0.6*μm* for most of the mushrooms, never exceeding 1.0*μm*. The right-hand SEM image shows the spores with a higher resolution. These spores were collected in summer 2017 and have a perfect spherical shape. The complex surface structure of the spores is thought to be responsible for hydrophobic properties [33].

Fig 2B displays the PSL microspheres that also exhibit a uniform size distribution. The mean size of these PSL particles was 3.5 *μm* (according to manufacturer: from 3 to 5 *μm*). The right-hand SEM image shows the PSL spheres similar in size to the spores, but their surface is smoother. Adhesion can be noticed between the PSL particles, which, as we show further on, have a tendency to agglomerate during storage and coagulate in the aerosol state.

Fig 2C depicts Al_2_O_3_ particles. It is clear from the macroscopic examination that the particles are very diverse in size. Statistical measurements resulted in (*D*_90_ − *D*_10_)/2 = (7.2*μm* − 2.4*μm*)/2 = 2.4*μm*. Nevertheless, this material has been accepted by some metrological organizations as a polydisperse reference material [18]. Hereafter, we shall compare other properties of the Al_2_O_3_ aerosol (i.e. aggregation, atomization convenience, etc.) with the puffball spores to show that the bioaerosol proposed here is at least as good as the other certified reference standards.

### Particle size change over time

The particle size stability in the course of the experiment is an important property of aerosols as it imposes limitations on the experiment duration. In some practical applications, such as instrument calibration [34], the stable particle distribution is required. The best option is when particles do not coagulate nor evaporate, and preserve the initial size (which can be controlled by other methods, e.g. microscopy) during the calibration process. As a result, the particle size would also be independent on the ambient conditions such as humidity, temperature, particle concentration, etc. that influence coagulation/evaporation.

The time course of the particle size distribution was examined by the Malvern Spraytec analyzer. The fungal spores were atomized by the air spray gun into a 0.5 m × 0.5 m × 0.5 m chamber. We have measured the statistical characteristics of the particle distribution function of the fungal spore bioaerosol over 14 minutes, and immediately after atomization the distribution featured the following statistical characteristics:

- arithmetic-mean particle diameter *D*(1, 0) = 3.61 *μm*;
- smallest particle diameter *D*_10_ = 2.23 *μm*;
- largest particle diameter *D*_90_ = 4.37 *μm*;
- specific surface area (S.S.A) = 1.62 *m*^2^/*cm*^3^;
- mean Sauter diameter *D*_32_ = 4.53 *μm*.

The raw measurement data for the particle size distribution (also for Al_2_O_3_ and PSL aerosols) can be found in S1 Appendix.

The mean Sauter diameter was chosen as the basic typical size because it characterizes the mass exchange between the particle and the environment. The mean Sauter diameter can be defined as the size of an imaginary sphere that shares the same volume/surface ratio with the entire ensemble of aerosol particles. The mean Sauter diameter is always larger than arithmetic-mean particle diameter *D*(1, 0), but the closer their values are, the narrower is the particle size distribution.

**Fig 3:**
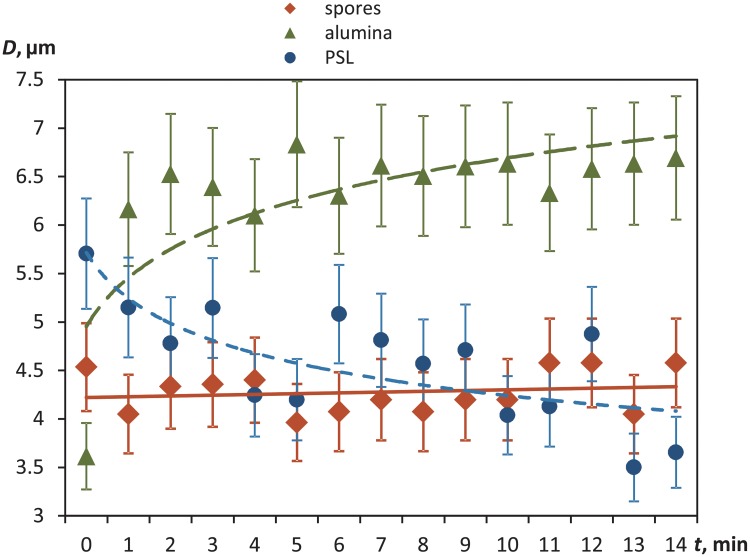
The time course of the mean Sauter diameter (*D*_32_) of the fungal bioaerosol particles (orange diamonds) compared to Al_2_O_3_ (green triangles) and PSL (blue circles). The trends of changes in the particle sizes are indicated by solid line (spores) and dashed line (alumina and PSL).

Fig 3 shows the time course of the mean Sauter diameter during 14 min post-atomization. The Al_2_O_3_ aerosol particles (green triangles) initially had *D*_32_ = 3.57 *μm*, and then the mean Sauter diameter almost doubled (up to *D*_32_ > 6 *μm*) within the first minute of the experiment, indicating the particle coagulation.

The mean Sauter diameter of the PSL spheres (Fig 3, blue circles) decreased over 14 minutes from *D*_32_ = 5.7 *μm* to 3.7 *μm* (30%). The initial *D*_32_ was larger due to water droplets that carried PSL particles, which then evaporated during the course of experiment. The size of the fungal spores at 14 min of observation was close to that of the PSL spheres measured in the microscope, that is 3.6 *μm*. The fungal spore size kept stable throughout the experiment (Fig 3, orange diamonds), showing only slight fluctuations (∼ 0.2*μm*) of the mean Sauter diameter. The fungal spores, as opposed to the Al_2_O_3_ particles and PSL spheres, remained stable for the whole 14 min of the measurements, suggesting that spores did not coagulate nor settled during the experiment.

**Fig 4:**
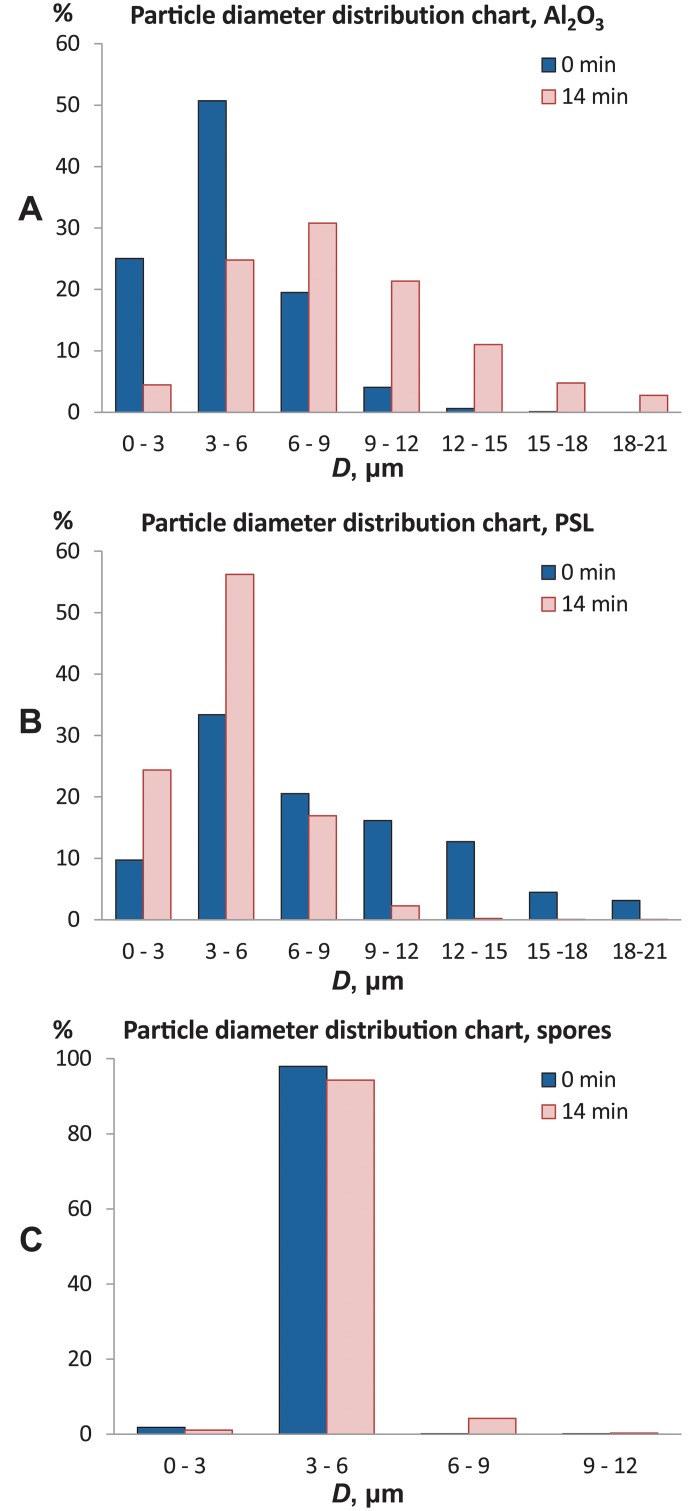
The particle size distribution immediately after atomization (blue) and after 14 min (pink) in the air. (A) Al_2_O_3_ particles. Distribution has shifted towards larger particle sizes, indicating that coagulation has occurred. (B) PSL microspheres. The size distribution narrowed and the mean Sauter diameter decreased because the water covering the spheres had evaporated. (C) Fungal spores. The size distribution showed no considerable change, suggesting that nor coagulation, nor settling had occur.

The particle size distributions at the beginning versus the end of the experiment are given in Fig 4. The broad Al_2_O_3_ size distribution (Fig 4A) and the increased Sauter diameter indicate coagulation. The PSL particle size distribution (Fig 4B) was broad at the beginning of the experiment due to the fact that water droplets carrying the PSL spheres had variable dimensions. But once the water had evaporated, the distribution got narrower and the mean Sauter diameter approached to the actual diameter of the microspheres. The fungal spore size distribution (Fig 4C) did not change considerably.

The fact that the fungal bioaerosol kept stable and no coagulation occurred can be explained by the electrostatic charge present on fungal spore surface. This charge prevents the spores from coagulation and coalescence [35]. The electrostatic charge and its relaxation time for 31 fungal species from basidiomycota division were measured by Saar et al. [29]: the charge for the spores in the aerosol state was in a range of 21—981 electron changes (or (0.3 − 16.0) ⋅ 10^−17^*C*) and decreased sevenfold within 47 minutes [29]. The surface charge measurement particularly for *L. pyriforme* was beyond the scope of this study.

We have also shown, that spores do not absorb water from the environment and their size is independent of the ambient humidity. To prove this, we exposed the spores to high (100%) humidity for 2 hours and measured the sizes of fungal spores before and after the exposure. Neither the mean diameter, nor the width of the size distribution changed (see Fig D in S1 Appendix). This fact gives us confidence in proposing the use of spores for device calibration at any air humidity conditions.

### Long-term storage

We have been collecting *L. pyriforme* mushrooms since 2014, and samples of the years 2014, 2016 and 2017 were dried and stored at room temperature in the laboratory. The fungal spores gathered in different years were compared in the early 2018.

**Fig 5:**
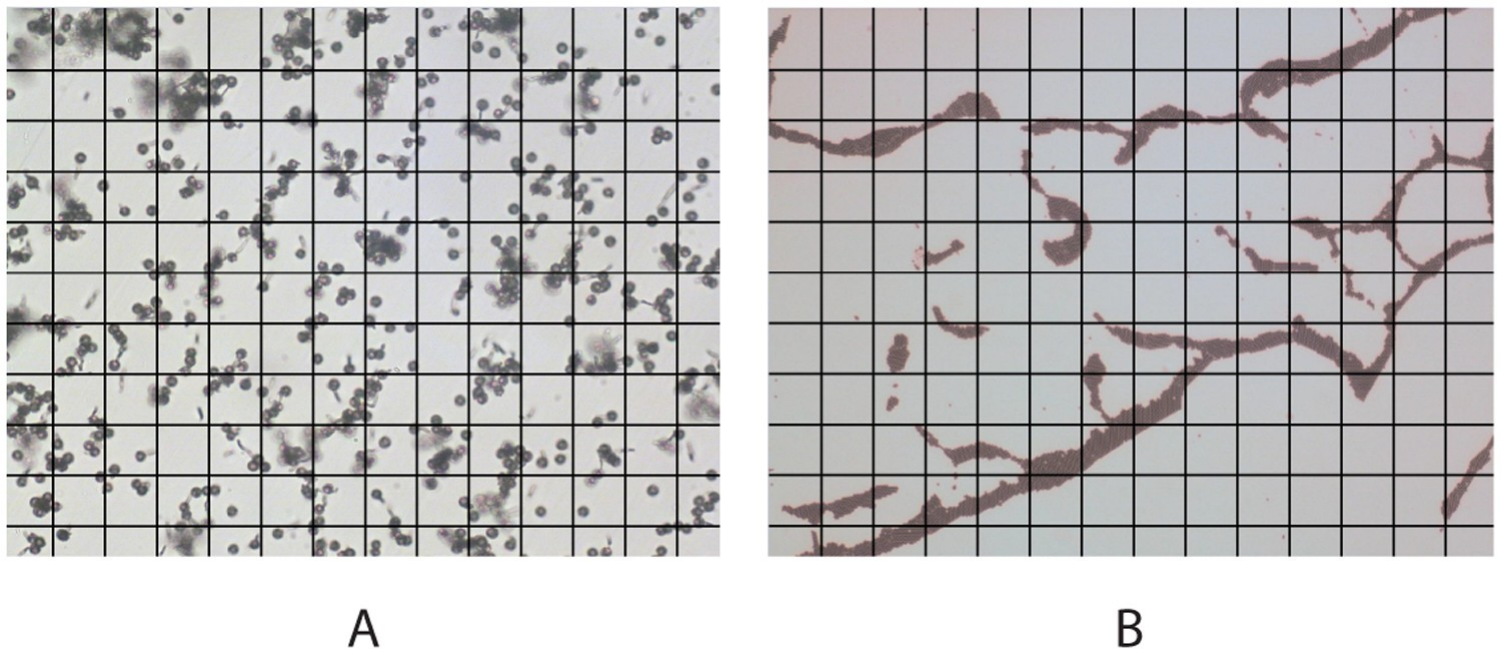
Storage-related changes in morphology of the fungal spores and PSL microspheres. (A) Spores after long-term storage (3.5 years). Some spores (about 20%) collected in 2014 had germinated, which, however, had no considerable effect on the size distribution (see Fig 6A). (B) PSL spheres after evaporation of the water solution aggregated into long chains.

After 3.5 years of storage, some fungal spores had germinated (Fig 5A). The spores gathered in 2016 did not show noticeable germination even in the mid-2018, two years post-collection. The fruiting bodies of all mushrooms, even of those gathered in 2014, were still functional as aerosol sprayers.

**Fig 6:**
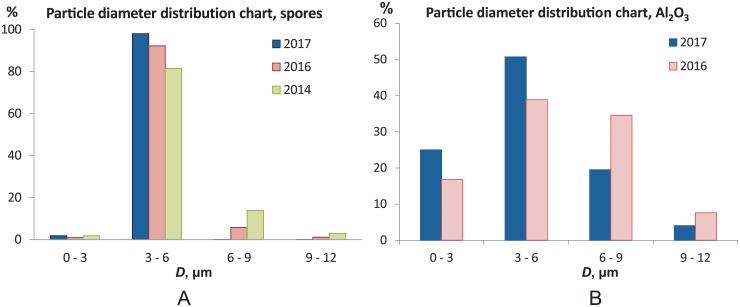
Size distributions of the particles collected/purchased at different times. (A) The size distribution of the spores of *L. pyriforme* collected in different seasons (2014, 2016, 2017). Spores from 2014 (green) had germinated and, therefore, the size distribution slightly shifted towards larger sizes. (B) The comparison of alumina powders purchased in 2016 and 2017. The particles that were stored 1 year longer had more stable aggregates and the size distribution shifted towards larger values.

Finally, we compared the particle size distributions of the spores collected in different years prior to germination (Fig 6A). We assume that these mushrooms produced spores of the same size each season, and, therefore, the difference in the spore sizes reflects the time evolution of such samples post-collection. Note that the fungi used in these experiments were collected in the same forest (N52°31.48′ E85°25.12′) in August-September each year, and belonged to the same species by their visual appearance. The size distributions show no difference between the samples collected in the 2017 and 2016 seasons (examined in the early 2018). However, the size distribution of the spores collected in 2014 shifted towards larger sizes. This is explained by the fact that some of these spores (approx. 20%) had germinated. So, it can be concluded that the spores and the fruiting bodies can be stored under normal conditions for at least 2 years, and this period can possibly be further extended by cold and dry storage.

The related statistics for the particle size measurements is provided in S1 Table. It summarizes the measurements from 8 different mushrooms studied with optical microscopy (with *n* > 700 spores segmented for each sample) and 24 mushrooms studied with Malvern Spraytec in the aerosol state. For each mushroom, the origin and the date of collection is specified.

The PSL spheres also have a shorter storage period, which is claimed to be 1 year by the manufacturers (All-Russian Research Institute of Metrological Service, and Applied Physics Inc.). This is essentially due to polystyrene ageing: polystyrene degrades and styrene dissolves into water, modifying the shape and size of the microspheres [19]. The other important reason is that the suspension dehydrates. Fig 5B shows the PSL spheres after the water solution has dried out. The particles aggregated and formed long chains. The particles could, however, be re-suspended from this state. Fig 5B also illustrates why the PSL spheres tend to coagulate in the aerosol: the water solution carrying the particles quickly evaporates once they are atomized, and the remaining dry particles produce aggregates [20].

Alumina particles can be stored for longer periods. They do form agglomerates during storage, but they are supposed to be atomized by ultrasound before each experiment. The distributions in Fig 6B suggest that the longer the powder is stored, the larger are the aggregates. The manufacturer sets a 5-year storage limit because agglomeration becomes irreversible by that time.

## Conclusion

We have compared spores of *Lycoperdon pyriforme* with two different, commonly used reference aerosol samples:

1. Polystyrene latex (PSL) microspheres were chosen as a reference sample of the monodisperse solid aerosol. The fungal spores and the PSL spheres had almost the same size (3.3 *μ*m diameter of spores vs. 3.5 *μ*m of PSL microspheres) and a similar circular shape (Fig 2).
2. Al_2_O_3_ particles were chosen for benchmarking as a certified reference standard for calibration of aerosol measuring instruments.
  This study showed that spores exhibit a much narrower size distribution, which expands their potential application range as a reference sample (especially for basic science). Secondly, the fungal bioaerosol remained stable for over 10 minutes, whereas the Al_2_O_3_ aerosol aggregated and settled as fast as within 1 min after atomization. The slow coagulation rate of the spores can be explained by their electrostatic properties [29]: the electric charge on their surface causes the spore particles to repulse from each other.

Thus, the spores of *L. pyriforme* and possibly other puffball fungi can be used as a reference sample of monodisperse aerosols that outperforms artificial reference samples such as Al_2_O_3_ powder and PSL microspheres. This biomaterial is widespread in nature, cheap in production, and also has a long shelf life, as demonstrated in this study. A more detailed and illustrative comparison of the three aerosol samples studied here is given in Table 1.

**Table 1:** Characteristics of different particles commonly used as standard aerosol samples compared to the spores of *Lycoperdon pyriforme*.

Criterion	Spores of *L. pyriforme*	Al_2_O_3_	PSL microspheres
Size distribution	Almost monodisperse (*D*_50_ = 3.6 *μm* for *L. pyriforme*)	Wider distribution of sizes (typically, *D*_50_ = 5 − 8 *μm*)	Nearly monodisperse (typically produced with *D*_50_ = 3 − 5 *μm*)
Morphology	Spheres	Shattered pieces with irregular shapes	Spheres
Aggregation during dry storage	No	When exposed to air, particles aggregate and form strong agglomerates	In suspension, spheres do not agglomerate but agglomerate quickly when exposed to the air
Coagulation in aerosol state	Do not coagulate for 15 min. Size distribution does not change	Coagulate in a few seconds, the size almost doubles (Fig 3) and the distribution widens	Coagulate rapidly [20] to form multiplets, which produce a discrete size distribution with distinct peaks [37]
Atomization convenience	Mushroom spore-bearing fruiting body is a natural aerosol generator	A sprayer and preliminary ultrasonic breakdown of agglomerates required	A special generator required [20]
“Model range”: size diversity	The size may slightly vary between different species of the *Lycoperdon* genus	A wide size range, from nanodisperse particles to the diameter of hundreds of micrometers	A wide size range, from nanodisperse particles to the diameter of tens of micrometers
Price per 10 g	$\tilde{\$}20$ (spores) https://www.amazon.com/Puffball-Mushroom-Langermannia-gigantea-Mycelium/dp/B01KLZ78FS ≈$0.2(whole fruiting body) https://sites.google.com/site/foragingct/home/price-list	$\tilde{\$}140$ http://granat-e.ru/catalog_moigms.html	$\tilde{\$}240$ http://www.magsphere.com/Products/Polystyrene-Latex-Particle/polystyrene-latex-particle.html
Shelf life	At least 2 years	Up to 5 years	1 year

Positive characteristics that facilitate calibration of optical analyzers of the aerosol particle size distribution are highlighted with a green background and negative ones with red.

## Discussion

This study introduces the spores of *Lycoperdon pyriforme* as a reference sample which, as the experiments have shown, outperforms artificially produced counterparts in many aspects. This bioaerosol is monodisperse and highly stable, has a long shelf-life time and low cost. Fungal spores were previously reported as a cell-counting tool in biological experiments [36], but their exceptional aerosol properties have not been studied before. Overall, fungal spores can significantly facilitate research and development of aerosol measurement techniques, especially optical methods that are currently in high demand [17].

Importantly, spores excel all artificially produced standard samples in stability towards coagulation in the air (stable for more than 15 minutes), which is attributed to electrostatic repulsion. The aerosol stability is vital for mushroom reproduction, since a longer time spent in the air results in a longer distance the spores can travel. Therefore, collective aerodynamic properties of the spores were tailored by evolution, selecting those species which could spread their genetic material over larger areas [29]. The biologically induced electrostatic charge in the spores [29, 35] is just one of the many mechanisms that prevent aggregation and coagulation. The fungi have a variety of microbiological “tools” that serve for better aerosol stability, such as hydrophobins on the surface (for hydrophobicity) [33]. As a result, the fungal spores are expectedly superior to manufactured materials in anti-coagulation and anti-aggregation abilities and could be utilized as a ready-to-use biotechnology.

There are numerous potential uses of fungal bioaerosols. Firstly, they provide a unique model for a stable monodisperse aerosol. This model facilitates research on optical measurement techniques for aerosol particle size, helping refine the existing methods and test new ones. Sedimentation of electrically charged bioaerosols is also an important research area. Natural aerosols are rich in electrically charged particles; thus, developing the techniques for their precipitation can potentially provide means for improvement of the air quality through air purification. Secondly, the size of *L. pyriforme* spores falls within the PM10 group being 3.6 *μm* in diameter. Therefore, the spores can be used as a solution to calibration of many PM10 measuring stations installed worldwide [6].

### Limitations

Since the fungal spores fall within the PM10 group, safety limitations apply to their use. When inhaled, the spores may infiltrate into the lung tissue causing irritation and inflammation [3], allergic responses, and can even lead to cancer in a long-term perspective [4]. The spores are also a bioactive material. The proteins on their surface may cause allergies and intoxication (if exposure is very intense) for certain people or animals [38]. Hydrophobins that provide the anti-coagulation coating for the spores are also considered as potential allergens [39]. The intense exposure to the spores may lead to a certain type of hypersensitivity pneumonitis called *Lycoperdonosis*. However, the existing safety standards prescribe the operator to wear a respirator during experiments, since all submicron particles, regardless of their chemical compound, may be hazardous to the operator. Thus, the standard safety precautions applied to experiments with submicron particles will be sufficient to protect the operator from Lycoperdonosis as well.

The other concern is that the spores maintain their fertility over a long period of time and can germinate and contaminate the area where the bioaerosol is used.

To avoid the aforementioned limitations, further research is needed. The spores can be chemically or genetically modified to suppress their fertility, and the biologically active proteins on their surface can be removed. The particle size-related safety precautions, however, persist, but the development of sedimentation techniques for bioaerosols that do not coagulate and settle spontaneously can resolve this problem in the future.

The spores have higher light absorption coefficient than the PSL spheres in the visible spectrum (8% higher for λ = 658*nm*). It would be interesting to measure optical properties of the spores in more detail, e.g. to characterize refraction and absorption at different wavelengths. However, these measurements lay beyond the scope of this study.

## Supporting information

S1 Video Puffball fungus *L. pyriforme* sprays spores. The slow-motion video shows (5x slowdown) how the puffball fungus sprays spores. Mushrooms were gathered in the 2014 (S1a), 2016 (S1b) and 2017 (S1c) seasons. The footage demonstrates the spraying process and how the puffball fruiting body generates an aerosol cloud.
(ZIP)

S1 Table Mean diameter *D*_1,0_ of the spores (left) and their Sauter diameter *D*_32_ in aerosol state (right). For each experiment, the origin of the mushroom and the date of collection are specified.
(DOCX)

S1 Appendix Source data on particle sizes. Output from OLYMPUS Particle Image Processor (PIP 9.0) software and Malvern Spraytec system, characterizing particle size distributions.
(DOCX)
