# Supplementary material for: Spores of puffball fungus Lycoperdon pyriforme as a reference standard of stable monodisperse aerosol for calibration of optical instruments
Source: PLoS One. 2019 Jan 30;14(1):e0210754. doi: 10.1371/journal.pone.0210754 (PMC6353166; doi:10.1371/journal.pone.0210754)
Supplement: S1 Table — For each experiment, the origin of the mushroom and the date of collection are specified. (DOCX) [file pone.0210754.s002.docx]

**S1 Table. Mean diameter *D*_1,0_ of the spores (left) and their Sauter diameter *D*_32_ in aerosol state (right).**

|  | **Optical microscopy** | | **Optical analyzer Malvern Spraytec** | | | | | |
| --- | --- | --- | --- | --- | --- | --- | --- | --- |
|  | **D_1,0_, μm** | |  |  | **D32, μm** | |  |  |
|  | Samples | |  |  | Samples | |  |  |
| **Collected** | 1 | 2 | 1 | 2 | 3 | 4 | 5 | 6 |
| 2018 y., autumn N52°48.45', E85°12.77' (Biysk) | 3.24 ± 0.98 (n=760) | 2.99 ± 0,90 (n=1240) | 4.81  (0.863) | 4.43  (0.701) | 3.93  (0.675) | 3.98  (0.866) | 4.53  (0.698) | 4.30  (0.785) |
| 2016 y., autumn N52°52.33', E84°72.62' (Sokolovo) | 3.34 ± 0.42  (n=791) | 3.44 ± 0.56 (n=836) | 4.4  (0.737) | 4.49  (0.700) | 4.54  (0.697) | 4.61  (0.668) | 4.31  (0.720) | 4.47  (0.679) |
| 2017 y., summer N52°31.48’ E85◦25.12’ (Malo-Eniseyskoe) | 3.34 ± 0.59 (n=722) | 3.43 ± 0.69 (n=736) | 4.06  (0.861) | 4.08  (0.847) | 3.97  (0.866) | 4.14  (0.838) | 4.47  (0.679) | 4.22  (0.746) |
| 2015 y., summer N52°31.48’ E85◦25.12’ (Malo-Eniseyskoe) | 3.20 ± 0.48 (n=842) | 3.35 ± 0.35 (n=940) | 4.73  (0.844) | 4.22  (0.746) | 4.33  (0.731) | 3.72  (0.870) | 4.06  (0.846) | 3.74  (0.845) |
| **Min - Max** | **2.99 - 3.43** | |  |  |  |  | **3.72 - 4.81** | |
| **Mean** | **3.29 (n_p_=6867, n_S_ = 8)** | |  |  |  |  | **4.28 (n_S_=24)** | |
| **Std/Mean** |  | **3,37 %** |  |  |  |  | **5.62 %** | |
