# Supplementary material for: Spores of puffball fungus Lycoperdon pyriforme as a reference standard of stable monodisperse aerosol for calibration of optical instruments
Source: PLoS One. 2019 Jan 30;14(1):e0210754. doi: 10.1371/journal.pone.0210754 (PMC6353166; doi:10.1371/journal.pone.0210754)
Supplement: S1 Appendix — Output from OLYMPUS Particle Image Processor (PIP 9.0) software and Malvern Spraytec system, characterizing particle size distributions. (DOCX) [file pone.0210754.s003.docx]

**S1 Appendix. Raw particle size data.**

**
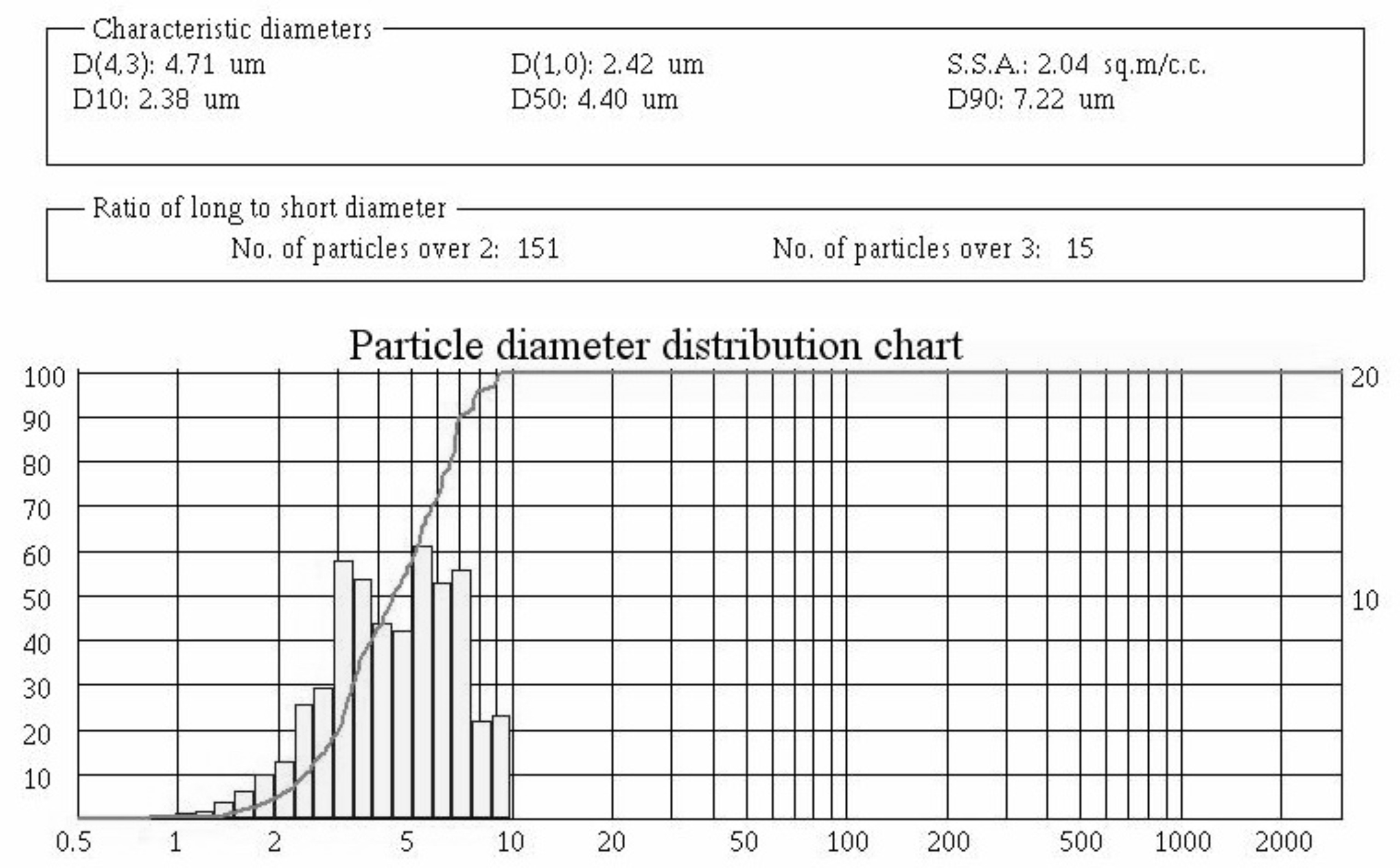
**

**(a)**

**
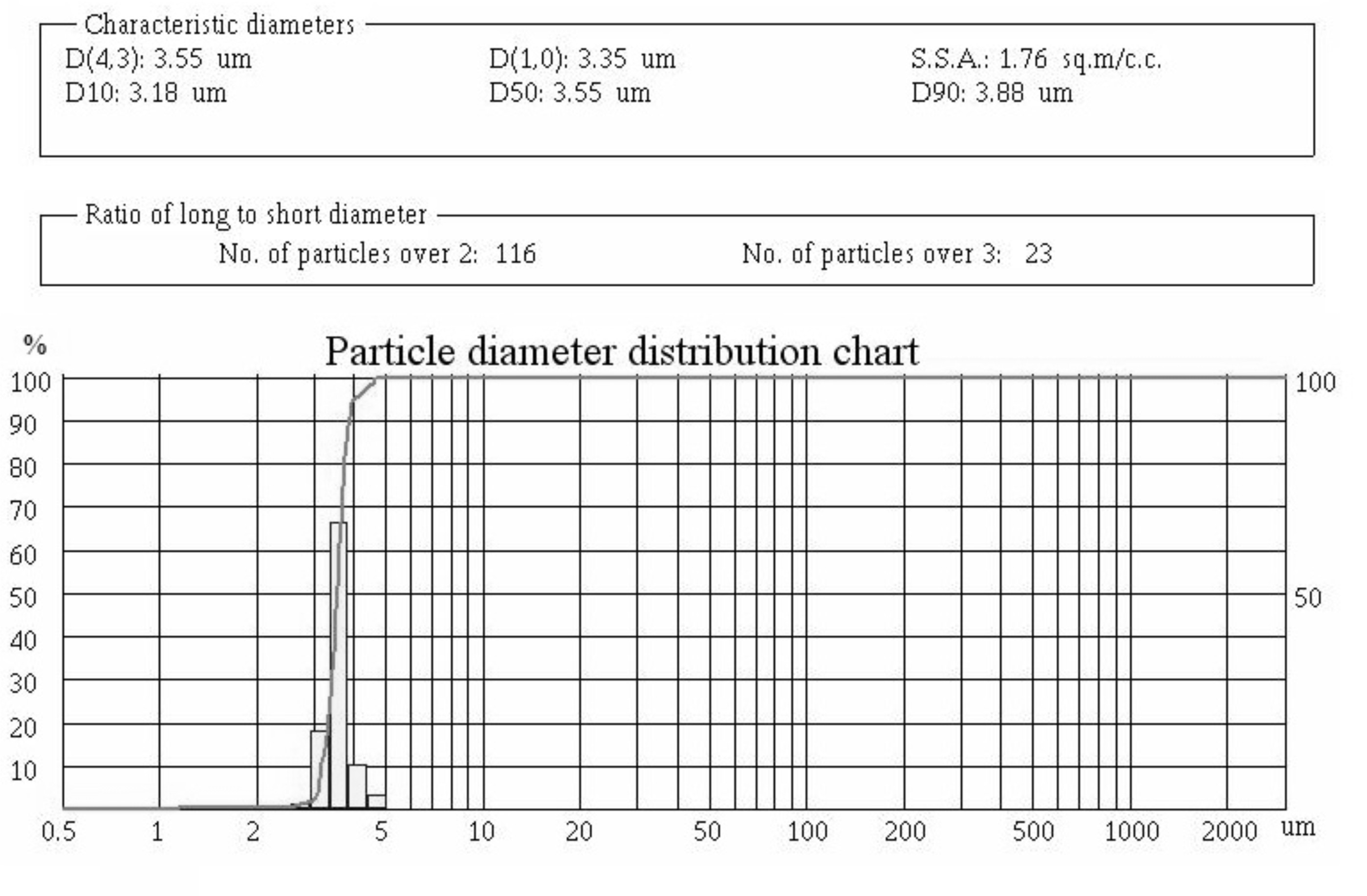
**

**(b**)


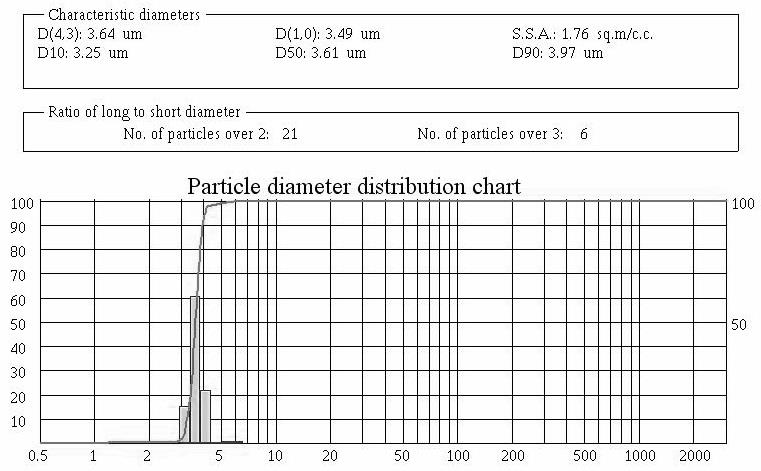


(c)

Fig A. Probability density function (PDF) and cumulative distribution function (CDF) for particle sizes of alumina powder (a), fungal spores (b), polystyrene latex (PSL) microspheres (c); measured with OLYMPUS OMEC DC130 and analyzed by OLYMPUS Particle Image Processor (PIP 9.0). Fungal spores and PLS microspheres have monodisperse distributions of particle sizes, and almost identical mean diameters (D(1,0) ≈ 3.5 μm). Alumina powder has a much wider distribution of particle sizes than fungal spores or PSL spheres.


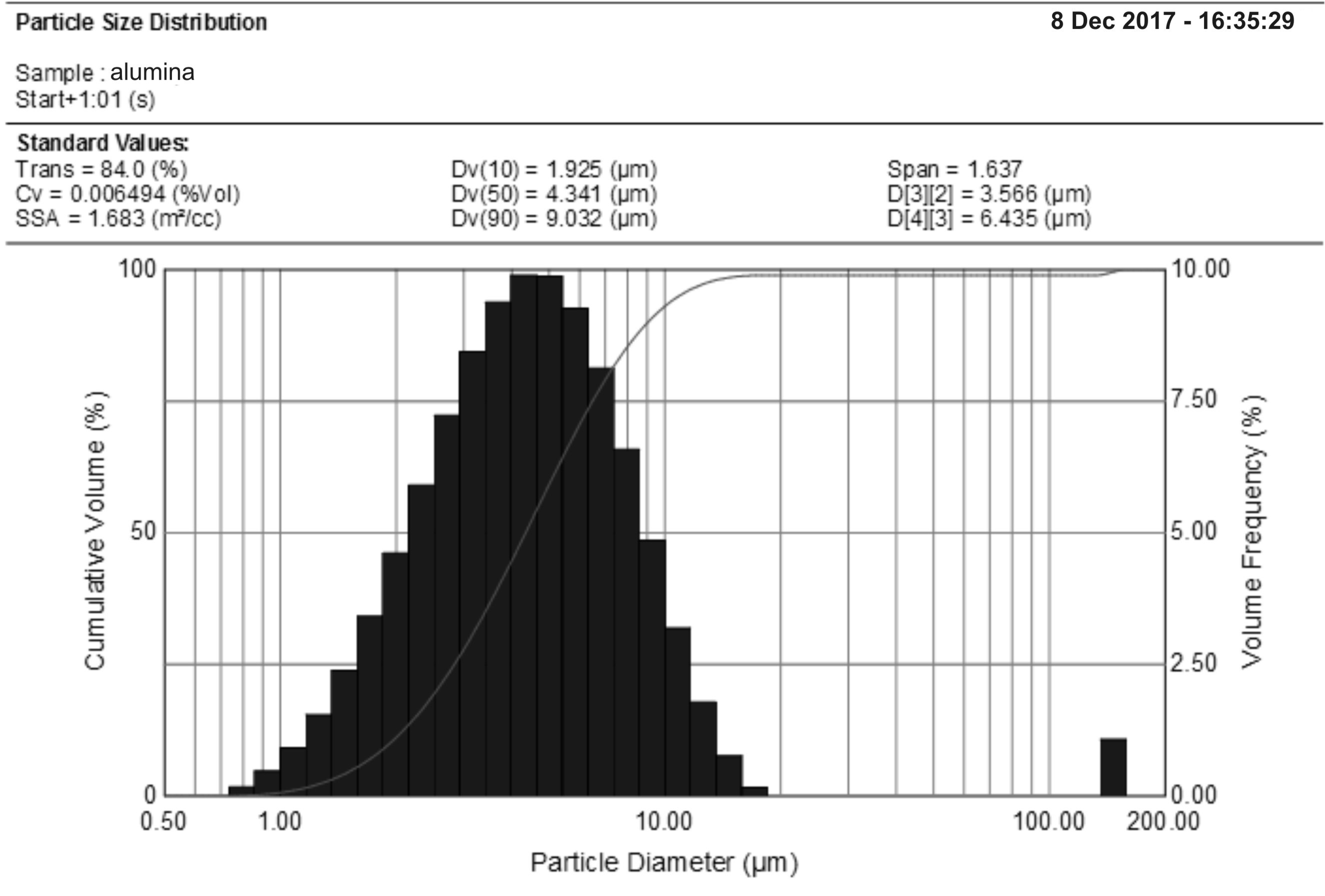


(a)


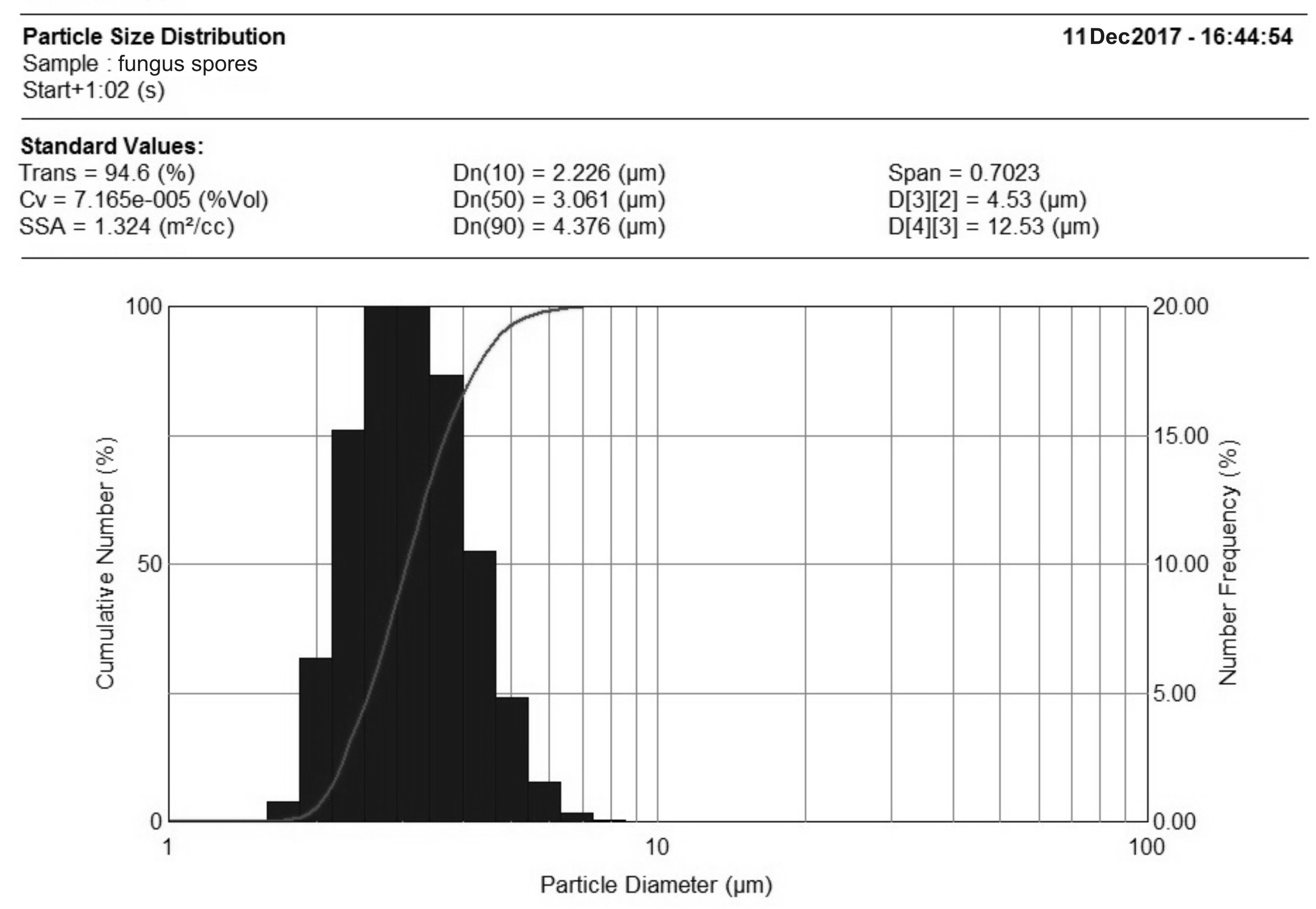


(b)


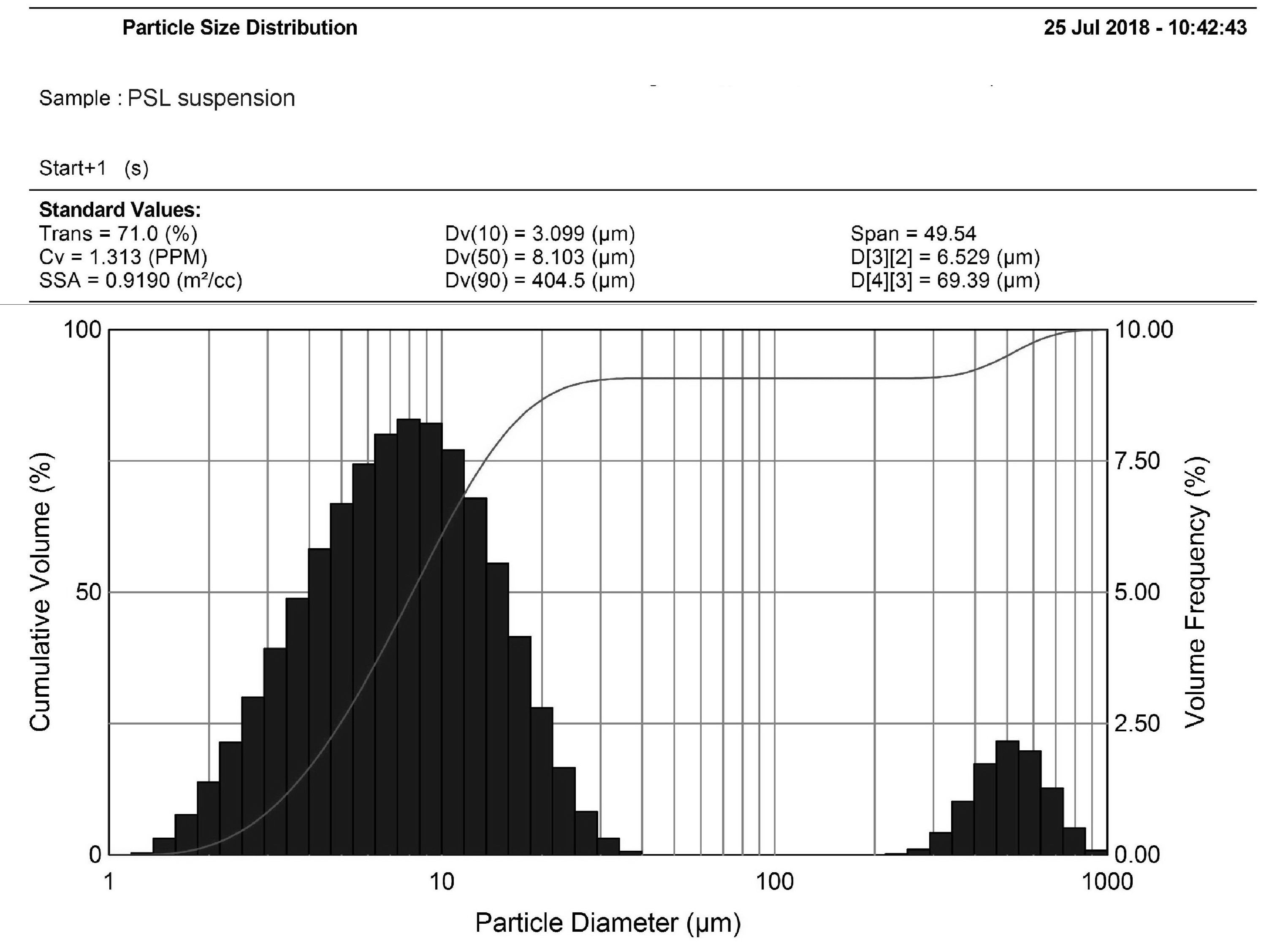


(c)


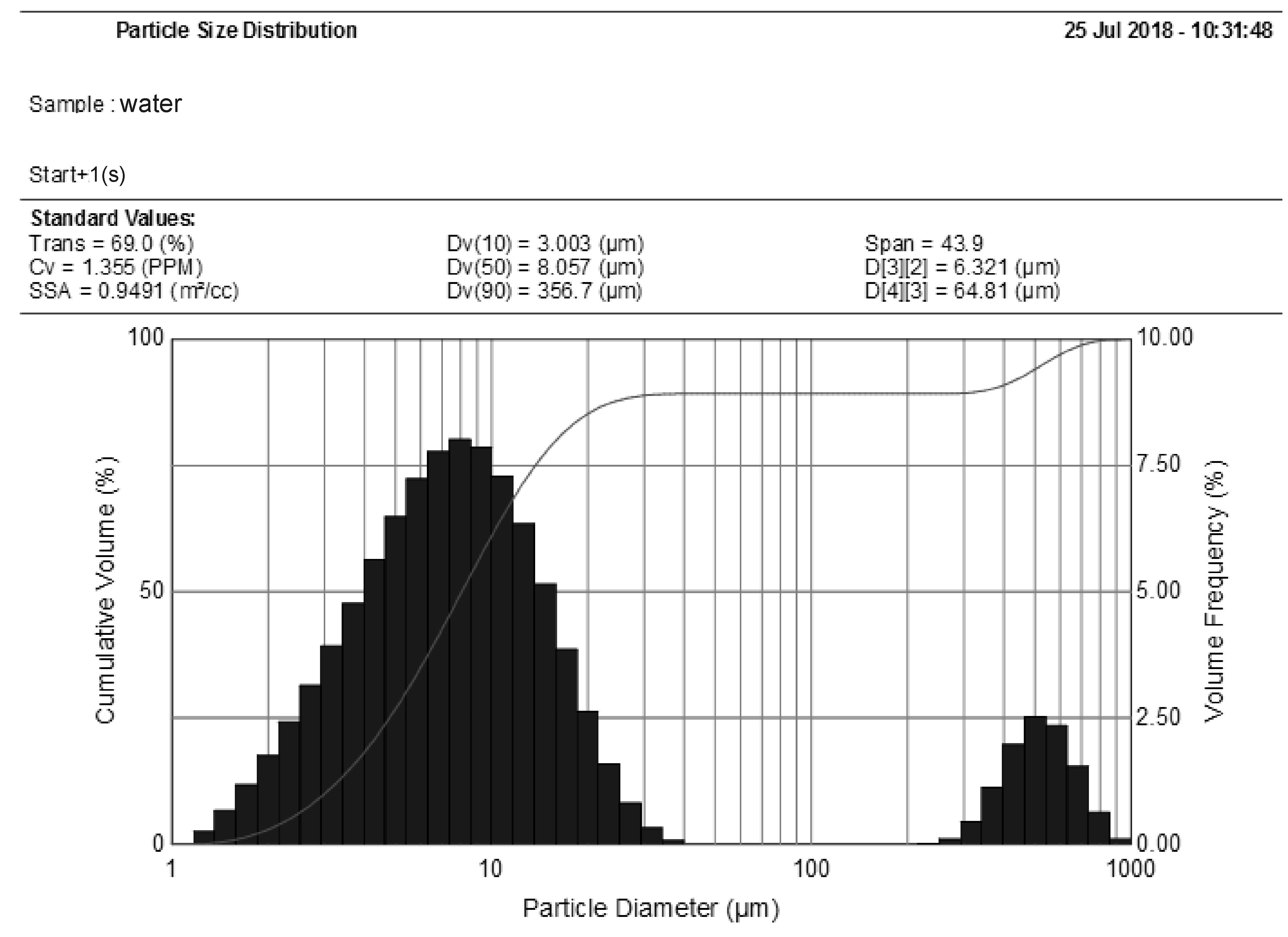


(d)

Fig B. Probability density function (PDF) and cumulative distribution function (CDF) of particle sizes of alumina (a), fungus spores (b), PSL microspheres (c); used in the aerosol state with the Malvern Spraytec system. Note, that PSL spheres were sprayed in water suspension, which results in an additional peak in the plot around 500 μm. Size distribution of water droplets sprayed in the same conditions is shown in (d). Contribution of the PSL spheres to the distribution is demonstrated in Figure 3.


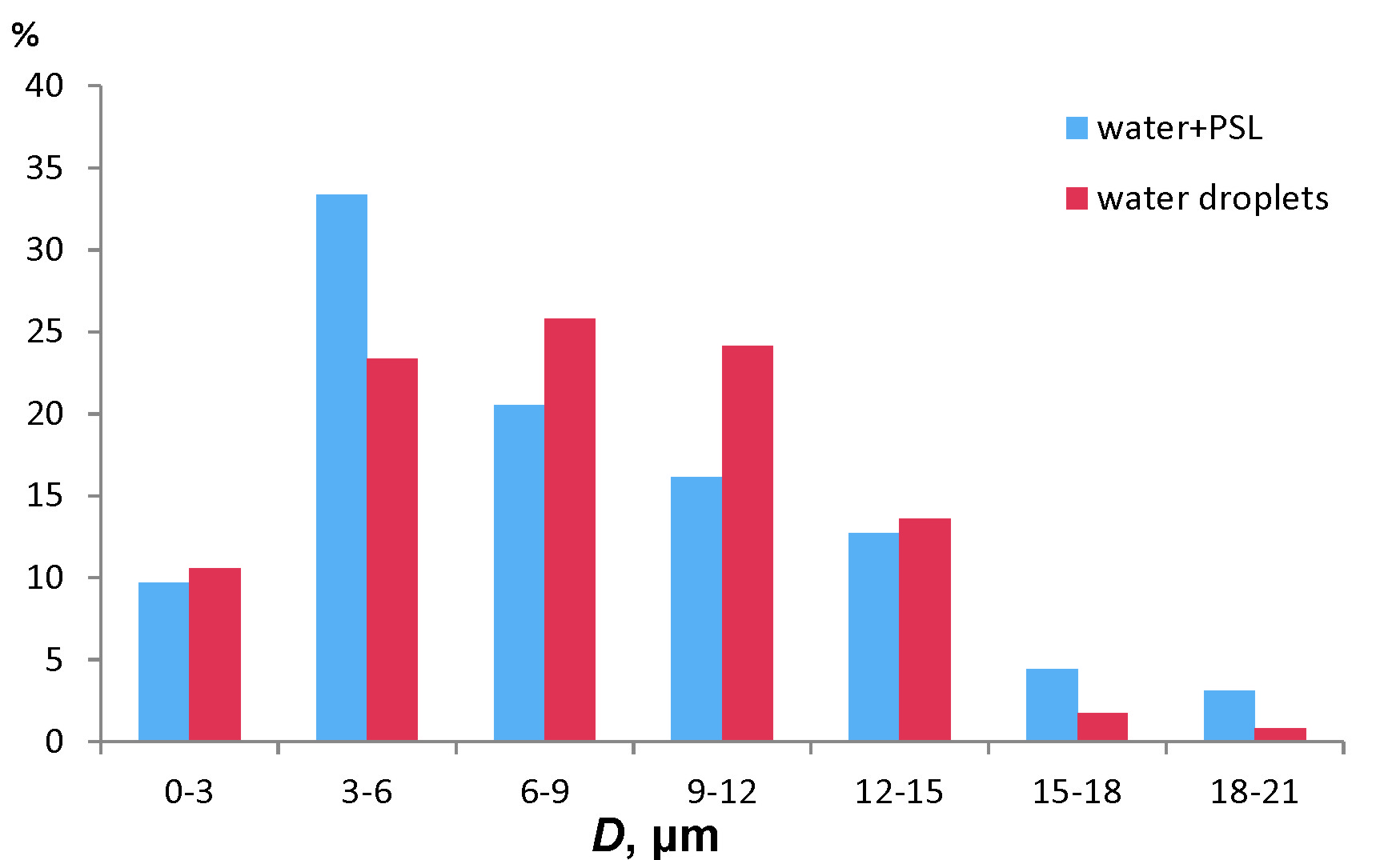


Fig C. Distributions of particle sizes of water droplets with and without PSL spheres plotted with wider bins **right after spraying**. This shows contribution of the PSL spheres (size ~4 μm) to the distribution.


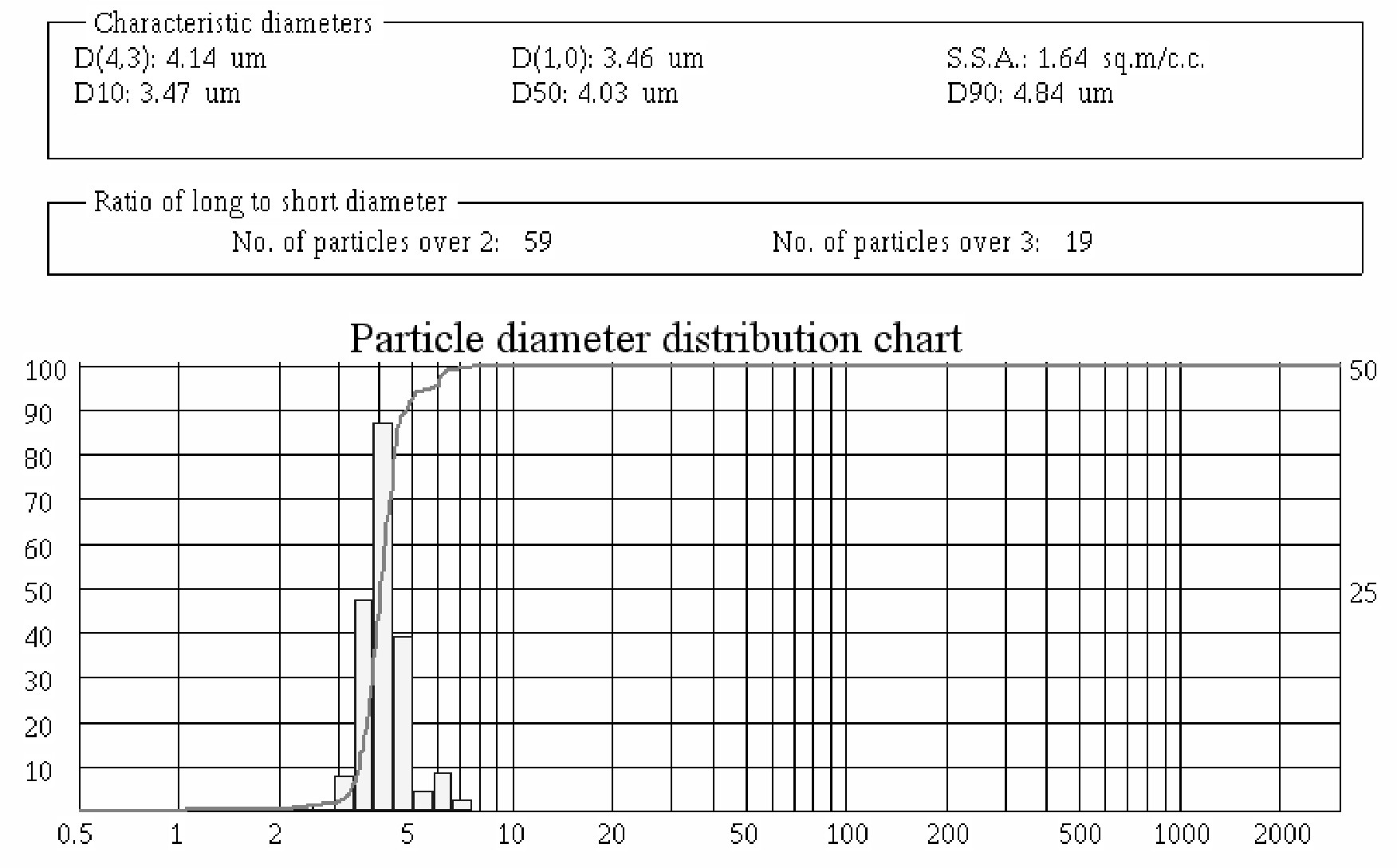


**(a**)


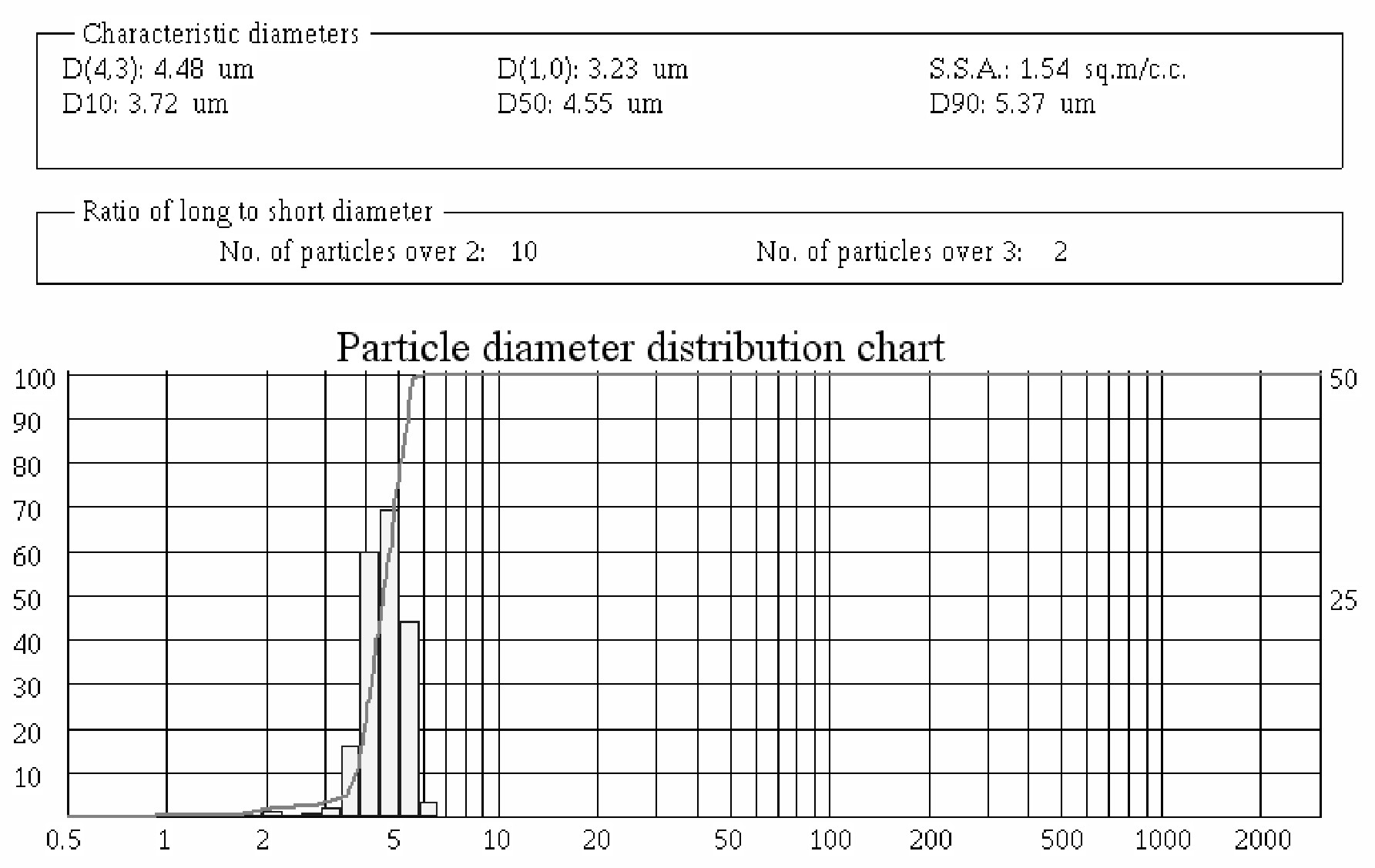


**(b**)

Fig D. Probability density function (PDF) and cumulative distribution function (CDF) for particle sizes of fungal spores: dry spores (a), wet spores (b). Spores were extracted from a mushroom collected in 2015 and the experiment was conducted in 2018.
